# Transcriptional profiles of different states of cancer stem cells in triple-negative breast cancer

**DOI:** 10.1186/s12943-018-0809-x

**Published:** 2018-02-23

**Authors:** Mingshan Liu, Yang Liu, Lu Deng, Dong Wang, Xueyan He, Lei Zhou, Max S. Wicha, Fan Bai, Suling Liu

**Affiliations:** 1 Biodynamics Optical Imaging Center (BIOPIC), School of Life Sciences, Peking University, No.5 Yiheyuan Road Haidian District, Beijing, 100871 China; 2 The CAS Key Laboratory of Innate Immunity and Chronic Disease, Hefei National Laboratory for Physical Sciences at the Microscale, School of Life Science and Medical Center, University of Science & Technology of China, Hefei, Anhui 230027 China; 3 Fudan University Shanghai Cancer Center & Institutes of Biomedical Sciences; Shanghai Medical College; Key Laboratory of Breast Cancer in Shanghai; Innovation Center for Cell Signaling Network; Cancer Institutes, Fudan University, Shanghai, 200032 China; 4 Comprehensive Cancer Center, Department of Internal Medicine, University of Michigan, Ann Arbor, MI 48109 USA

**Keywords:** Triple-negative breast cancer, Cancer stem cells, Whole-transcriptome sequencing

## Abstract

Breast cancer stem cells (BCSCs) are thought to be responsible for tumor initiation, metastasis and relapse. Our group and others have described markers useful in isolating BCSCs just as aldehyde dehydrogenase positive (ALDH^+^) or CD24^−^CD44^+^. In fact, cells which simultaneously express both sets of markers have the highest tumor initiating capacity. Although the transcriptomic profile of cells expressing each BCSC marker alone has been reported, the profile of the most tumorigenic population expressing both sets of markers has not. Here we used the biomarker combination of ALDH and CD24/CD44 to sort four populations isolated from triple-negative breast cancer (TNBC) patient-derived xenografts, and performed whole-transcriptome sequencing on each population. We systematically compared the profiles of the three states of BCSCs (ALDH^+^CD24^−^CD44^+^, ALDH^+^non-CD24^−^CD44^+^ and ALDH^−^CD24^−^CD44^+^) to that of the differentiated tumor cells (ALDH^−^non-CD24^−^CD44^+^). For the first time, we compared the ALDH^+^CD24^−^CD44^+^ BCSCs with the other two BCSC populations. In ALDH^+^CD24^−^CD44^+^ BCSCs, we identified *P4HA2*, *PTGR1* and *RAB40B* as potential prognostic markers, which were virtually related to the status of BCSCs and tumor growth in TNBC cells.

**Electronic supplementary material:**

The online version of this article (10.1186/s12943-018-0809-x) contains supplementary material, which is available to authorized users.

## Background

Triple-negative breast cancer (TNBC) is primarily identified through a lack of expression of estrogen and progesterone (ER and PR, respectively), and the gene ERBB2 (ER^−^PR^−^HER2^−^) [1]. TNBC is the subtype of breast cancer with the poorest clinical outcome and lack of targeted therapy [2]. Cancer stem cells (CSCs) [3], or tumor-initiating cells, are capable of self-renewal and differentiation, which are considered to be responsible for tumorigenesis and cancer relapse [4]. Eradication of breast cancer stem cells (BCSCs) may result in improved clinical outcomes.

It is common to use fluorescent activated cell sorting and specific biomarkers of BCSCs to isolate BCSCs from heterogeneous tumor tissues, patient-derived xenografts (PDXs) and cell lines [5–7]. BCSCs were widely recognized to be enriched with the biomarkers CD24^−^CD44^+^ [8] or ALDH^+^ [9]. Our previous studies have demonstrated cells expressing the biomarkers CD24^−^CD44^+^ and ALDH^+^ exist across all subtypes of breast cancer, although in varying proportions. Furthermore, we have demonstrated that BCSCs in the mesenchymal state are characterized as CD24^−^CD44^+^ BCSCs, while ALDH^+^ BCSCs are characterized as epithelial [7]. In breast cancer, ALDH^+^CD24^−^CD44^+^ cells are rare population within tumors and cell lines, which are endowed with greatest tumorigenesis and invasive capacity. ALDH^+^CD24^−^CD44^+^ cells can generate tumors in NOD/SCID mice, showing the greatest tumor-initiating capacity [9]. We postulate here that the ALDH^+^CD24^−^CD44^+^ cells are more purified BCSC population. Here we used the biomarker combinations ALDH and CD24/CD44 to divide cells from two TNBC PDXs into four groups to systematically compared different states of BCSCs on transcriptome to get potential prognostic genes in TNBC.

### Findings

#### Transcriptional analysis between three states of BCSCs and the differentiated tumor cell population

**Fig. 1:**
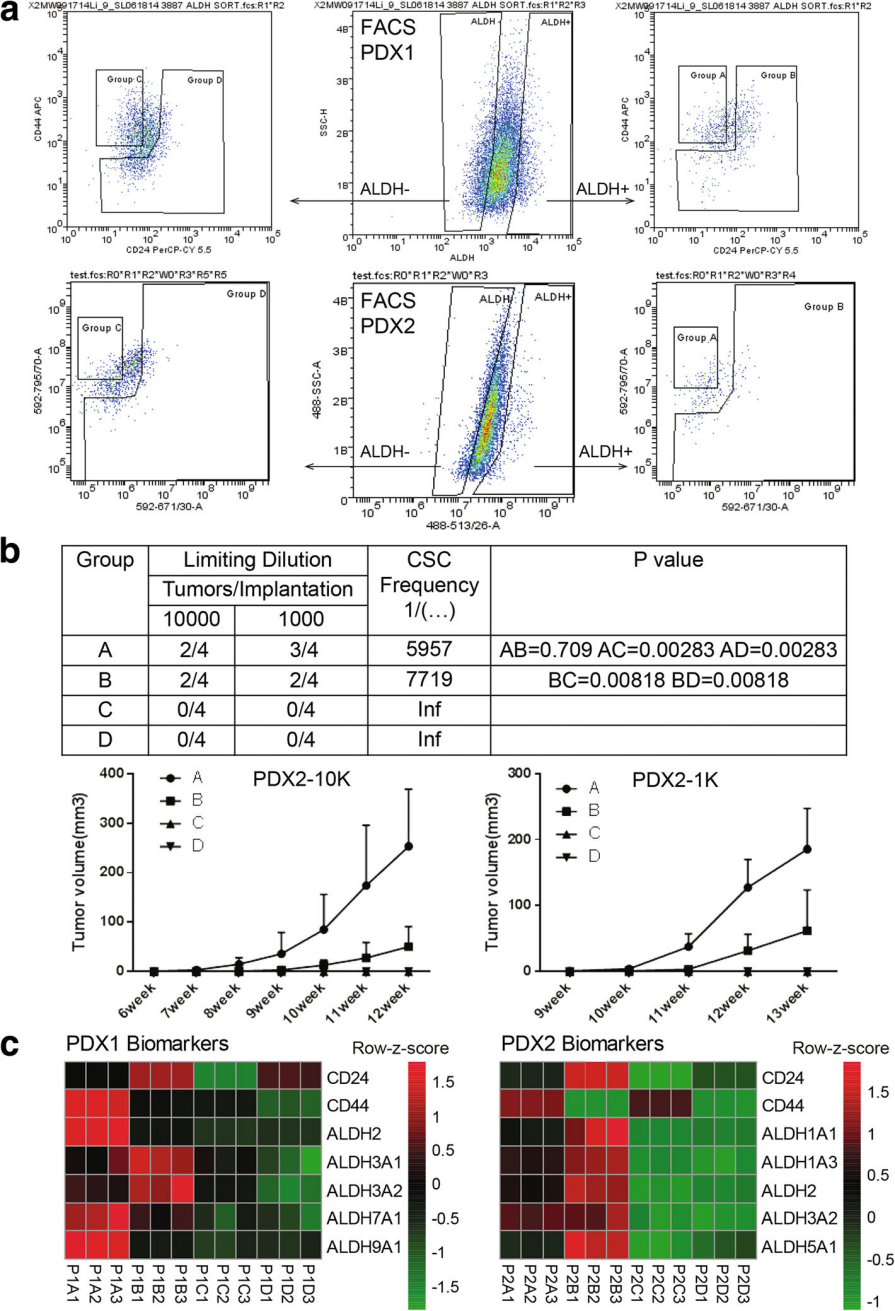
Isolation and characterization of the four cell populations from PDXs. (**a**) The flow charts of ALDH, CD24 and CD44 for PDX1 and PDX2 by fluorescent activated cell sorting. We isolated four groups based on biomarker combinations of ALDH and CD24/CD44. (**b**) The limiting dilutions of cells obtained from PDX2 (VARI068) were implanted in the fourth fat pads of NOD-SCID mice. The tumor growth for each group was monitored and calculated weekly, and the CSC frequency for the group A, B, C, D was calculated based on the website http://bioinf.wehi.edu.au/software/elda/. (**c**) The expression of BCSC biomarkers ALDH, CD24 and CD44 in each sorted group. We compared each group with the following criteria: 1) CD24: A < B, C < D; 2) CD44: A > B, C > D; 3) ALDH: A/B > C/D. PDXs have different expressions of ALDH isoforms. P1: PDX1; P2: PDX2

To systematically characterize the transcriptional profiles of BCSCs, we isolated four cell groups from two TNBC PDXs, and performed whole-transcriptome sequencing to identify differentially expressed genes (DEGs) between four groups (Fig. 1a): (1) group A (ALDH^+^CD24^−^CD44^+^, highly purified BCSCs); (2) group B (ALDH^+^non-CD24^−^CD44^+^, enriched epithelial-like BCSCs); (3) group C (ALDH^−^CD24^−^CD44^+^, enriched mesenchymal-like BCSCs); and (4) group D (ALDH^−^non-CD24^−^CD44^+^, differentiated tumor cells). The tumorigenicity of each cell population was analyzed in vivo, and the result demonstrated that groups A and B had significantly higher tumor-initiating capacity and CSC frequency than groups C and D (Fig. 1b), with the highest tumorigenicity for group A. Moreover, the size of tumors in group A was significantly larger than that in group B. The transcriptomic data is shown in Additional file 1: Table S1. The expression of BCSC biomarkers ALDH and CD24/CD44 were as expected [7]: CD24: A < B, C < D; CD44: A > B, C > D; ALDH: A/B > C/D (Fig. 1c). We systematically performed pair-comparisons between three subsets of BCSCs and differentiated tumor cells (Additional file 1: Figure S1) with fold change set at 1.2 based on the standard of our previous study [7]. The DEGs in A/D, B/D and C/D pair-comparisons were 3223, 3387 and 3065, respectively (Additional file 1: Figure S1a). For all states of BCSCs in common, there were 391 DEGs in the intersection set (Additional file 1: Figure S1b). The Gene Ontology (GO) analysis based on biological process indicated that the 391 DEGs involved in cellular response to hypoxia, cell adhesion, extracellular matrix organization, cell cycle, etc. (Additional file 2: Table S2). To characterize the exclusively transcriptional features of each state of BCSCs, we overlapped the DEGs of three pair-comparisons (Additional file 1: Figure S1b), and found that each state has its own unique DEGs (Additional file 1: Figure S1b), of which the altered GO terms were different identified by DAVID 6.8 and Gene Set Enrichment Analysis (GSEA) (Additional file 1: Figure S2, Additional file 3: Table S3), suggesting that three populations of the ALDH^+^CD24^−^CD44^+^, the ALDH^+^non-CD24^−^CD44^+^ and the ALDH^−^CD24^−^CD44^+^ were different states of BCSCs. In addition, we also found that the epithelial markers, CDH3, CLDN3, CLDN4, CLDN7 and MKI67, were highly expressed in the ALDH^+^non-CD24^−^CD44^−^ BCSCs, while the mesenchymal markers, CDH2, FOXC2, MMP2, SNAI2 and TWIST1, were highly expressed in the ALDH^−^CD24^−^CD44^+^ BCSCs (Additional file 1: Figure S3).

#### Comparison of the gene transcription between ALDH^+^CD24^−^CD44^+^ BCSCs and the other three groups

**Fig. 2:**
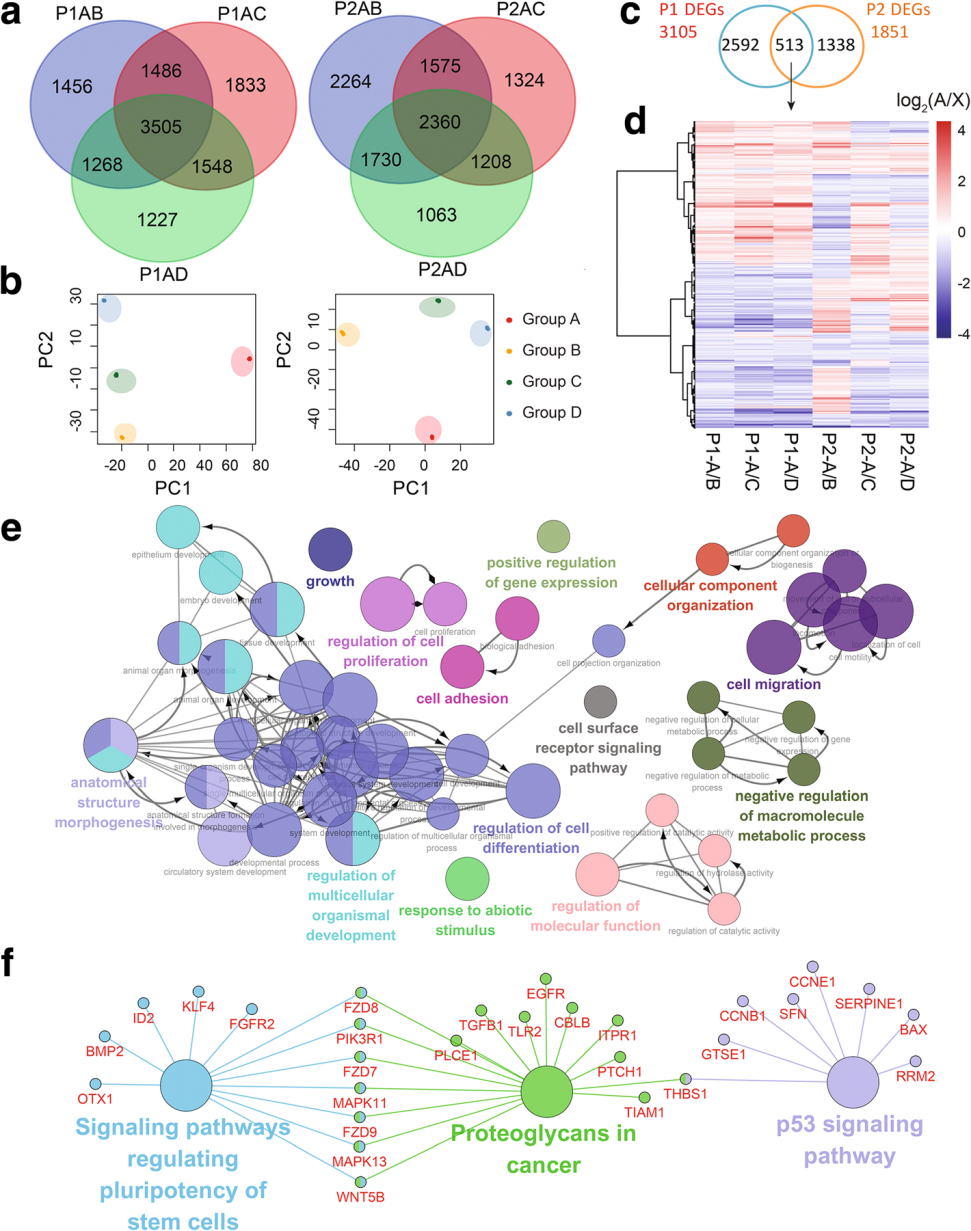
The unique DEGs of ALDH^+^CD24^−^CD44^+^ BCSCs. (**a**) The Venn diagrams of the DEGs between ALDH^+^CD24^−^CD44^+^ BCSCs (group A) and other three groups with fold change set 1.2. (**b**) The principal component analysis (PCA) plots of DEGs in two PDXs. (**c**) The intersection set of DEGs after filtered by PCA in two PDXs. (**d**) The intersected 513 DEGs in two PDXs. (**e**) The GO analysis based on biological processes of the 513 DEGs visualized by Apps ClueGO v2.3.2 of Cytoscape v3.4.0 with network specificity set Global. (**f**) The KEGG pathway analysis of the 513 DEGs visualized by Cytoscape v3.4.0 with network specificity set medium

To identify the DEGs in ALDH^+^CD24^−^CD44^+^ BCSCs, we compared group A with the other three groups with fold change set at 1.2 in analyzed PDXs (Fig. 2a). The numbers of intersected A/X (X stands for groups B, C or D) DEGs overlapped in analyzed PDXs were 3505 and 2360, respectively (Fig. 2a). We performed principal component analysis to further distinguish group A from the other three groups in each PDX, trimming DEGs to 3105 and 1851 for PDX1 and PDX2, respectively (Fig. 2b, c). Then we overlapped the trimmed DEGs of analyzed PDXs and identified 513 DEGs in the intersection set (Fig.2c, d). After analyzing the 513 DEGs by GO analysis and KEGG pathway analysis, we found that ALDH^+^CD24^−^CD44^+^ BCSCs differed from the other populations in p53 signaling pathway, signaling pathways regulating pluripotency of stem cells, and central carbon metabolism in cancer, etc. (Fig. 2e, f, Additional file 4: Table S4). GSEA of the 513 DEGs also showed that the process of differentiation and development in ALDH^+^CD24^−^CD44^+^ BCSCs was significantly downregulated (Additional file 1: Figure S2, Additional file 3: Table S3).

#### Identification of the potential prognostic genes enriched in ALDH^+^CD24^−^CD44^+^ BCSCs of TNBC

To obtain unique A/X DEGs (X stands for groups B, C or D), we identified 90 out of 513 DEGs in two PDXs, the 38 upregulated (A > X) and 52 downregulated (A < X) genes in common (Additional file 1: Figure S4a). The GO analysis based on biological process identified *PPIL3*, *P4HA2* and *FKBP2* from 38 upregulated genes were involved in peptidyl-proline modification, suggesting that there might be some epigenetic modifications exclusively in BCSCs, while 52 downregulated genes were involved in regulation of cell differentiation, positive regulation of developmental process, regulation of multicellular organismal development and regulation of cell development (Additional file 4: Table S4). To search for potential prognostic markers of TNBC, we used the Kaplan-Meier plotter [10] to screen the 90 DEGs identified from ALDH^+^CD24^−^CD44^+^ BCSCs in analyzed PDXs. Among the 90 DEGs of purified BCSCs in PDXs (Additional file 1: Figure S4a), the high expression of *P4HA2* (*n* = 255, *p* = 0.00057) and *PTGR1* (*n* = 161, *p* = 0.001), and low expression of *RAB40B* (n = 255, *p* = 0.0069) in TNBC patients were associated with decreased RFS (Additional file 1: Figure S4b).

#### Knockdown of potential prognostic genes affected the status of BCSCs

**Fig. 3:**
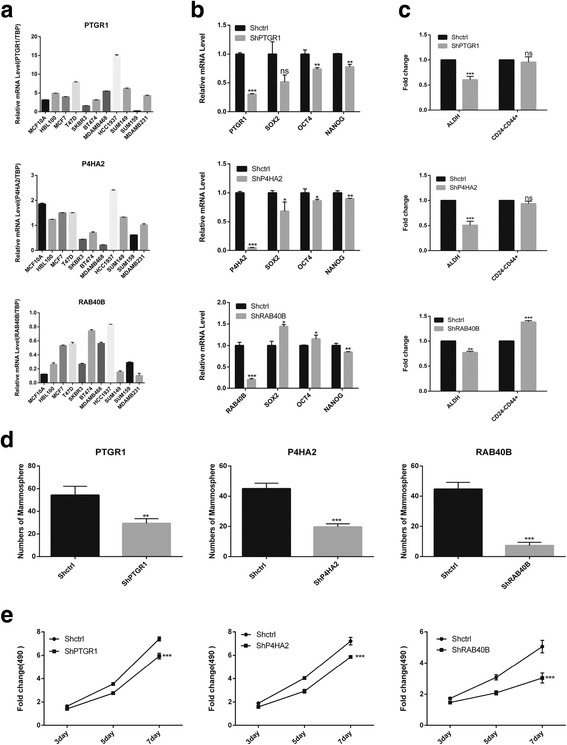
Functional analysis of potential prognostic genes. (**a**) The expressions of *PTGR1*, *P4HA2* and *RAB40B* was variable across different breast cell lines, including: 1) normal mammary gland cell lines, MCF10A and HBL100; 2) luminal breast cancer cell lines, MCF7 and T47D (ER^+^PR^−^HER2^−^); 3) HER2^+^ breast cancer cell lines (ER^−^PR^−^HER2^+^) containing SKBR3, BT474; 4) Basal-like/TNBC (ER^−^PR^−^HER2^−^) breast cancer cell lines, such as MDA-MB-468, HCC1937, SUM149, SUM159 and MDA-MB-231. (**b**) The expressions of CSC-related genes (ShPTGR1, ShP4HA2 and ShRAB40B) in the knockdown and the control (Shctrl) TNBC cell line SUM149. (**c**) The fold change for the proportion of each BCSC state in knockdown cells vs. Shctrl cells as assessed by fluorescent activated cell sorting. (**d**) The mammosphere formed in Shctrl cells and knockdown cells accessed by mammosphere formation assay. (**e**) The fold change for cell proliferation of knockdown SUM149 cells vs. Shctrl SUM149 cells as assessed by MTT assay . *, *P* < 0.05; **, *P* < 0.01; ***, *P* < 0.001; ns, not significant (compared with the corresponding Shctrl group). Error bars, mean ± SD

As assessed by quantitative real-time PCR (qRT-PCR), the relative expressions of *PTGR1*, *P4HA2* and *RAB40B* was variable across different breast cancer cell lines, for instance, the expression of *RAB40B* was comparatively lower in TNBC cell lines, such as SUM149, SUM159 and MDAMB231, than those of the other cell lines (Fig. 3a). To further elucidate the role of these genes in TNBC, we used shRNA to knock down each gene in TNBC cell line SUM149. The expressions of *PTGR1*, *P4HA2* and *RAB40B* were significantly lower after lentivirus infection confirmed by qRT-PCR (Fig. 3b). Knockdown of *P4HA2* or *PTGR1* downregulated CSC-related genes, such as *SOX2*, *OCT4* and *NANOG* (Fig. 3b), as well as causing a significant decrease in the proportion of BCSCs as assessed by ALDEFLUOR assay (Fig. 3c) and mammosphere formation assay (Fig. 3d). However, knockdown of *P4HA2* or *PTGR1* had no effect on CD24^−^CD44^+^ population of SUM149, but only on ALDH^+^ population (Fig. 3c). In addition to their effect on the BCSC population, knockdown of *P4HA2* or *PTGR1* also inhibited cell proliferation verified by MTT assay (Fig. 3e). When we knocked down *RAB40B*, the CSC-related genes, *SOX2* and *OCT4*, were upregulated (Fig. 3b). In addition to that, the amount of the mesenchymal-like BCSCs (CD24^−^CD44^+^) was increased (Fig. 3c). Interestingly, knockdown of *RAB40B* also prevented mammosphere formation (Fig. 3d) and cell proliferation in SUM149 (Fig. 3e). To further validate the function of RAB40B in TNBC, we used two different shRNAs (RAB40BSh-sh2 used in SUM149, and another new sequence RAN40BSh-sh3) to knockdown the expression of RAB40B in another two TNBC cell lines: SUM159 and MDA-MB-231. The shRNAs worked well as assessed by qRT-PCR (Additional file 1: Figure S5a). Knockdown of RAB40B up-regulated CSC-related genes, such as SOX2, OCT4 and NANOG (Additional file 1: Figure S5.a), consistent with the results in SUM149 (Fig. 3b). Knockdown of RAB40B had no effect on CD24^−^CD44^+^ population of SUM159 and MDA-MB-231 (Additional file 1: Figure S5b), however, knockdown of RAB40B significantly increased ALDH^+^ population (Additional file 1: Figure S5b), as well as causing a remarkable increase in mammosphere formation (Additional file 1: Figure S5c) and proliferation (Additional file 1: Figure S5d). These results seemed contradictory with the observation from SUM149, but this observation suggested RAB40B might play different roles in different cancer cells by affecting different BCSC population and also supported our previous report about the different proliferative capacity and cellular function between ALDH^+^ population and CD24^−^CD44^+^ population [7]. The functional analysis demonstrated that knockdown of the three potential prognostic markers would significantly affect the status of BCSCs and tumor growth simultaneously, indicating these genes might serve as the important prognostic markers in TNBC.

## Conclusion

This is the first transcriptional characterization of the ALDH^+^CD24^−^CD44^+^ BCSCs in TNBC, as well as the first comparisons between the ALDH^+^CD24^−^CD44^+^ BCSCs and other types of BCSCs in TNBC. In ALDH^+^CD24^−^CD44^+^ BCSCs, we identified three potential prognostic markers, *P4HA2*, *PTGR1* and *RAB40B*, which were related to the status of BCSCs and tumor growth in TNBC cells.

## Additional files

Additional file 1: Supplementary information including Materials and Methods, **Figure S1**-**5** and **Table S1 S5**. (DOCX 1332 kb)

Additional file 2: **Table S2.** GO analysis of 391 DEGs by DAVID 6.8. (XLS 35 kb)

Additional file 3: **Table S3.** GSEA summary. (XLSX 360 kb)

Additional file 4: **Table S4.** The KEGG pathway and GO analysis of 513 DEGs. (XLS 35 kb)

## Abbreviations

ALDH: Aldehyde dehydrogenase
BCSCs: Breast cancer stem cells
CSCs: Cancer stem cells
DEGs: Differentially expressed genes
GO: Gene Ontology
GSEA: Gene Set Enrichment Analysis
PDXs: Patient-derived xenografts
qRT-PCR: Quantitative real-time PCR
TNBC: Triple-negative breast cancer
