# Supplementary material for: Transcriptional profiles of different states of cancer stem cells in triple-negative breast cancer
Source: Mol Cancer. 2018 Feb 23;17:65. doi: 10.1186/s12943-018-0809-x (PMC5824475; doi:10.1186/s12943-018-0809-x)
Supplement: Supplementary file 1 — Supplementary information including Materials and Methods, Figure S1-5 and Table S1 S5. (DOCX 1332 kb) [file 12943_2018_809_MOESM1_ESM.docx]

## Supplementary information

## Materials and Methods

### Sample preparation and cell culture

Human breast tumors were obtained as biopsy cores or pieces of tumors after surgery and implanted into the fourth humanized cleared fat pads of NOD/SCID mice for establishing xenografts. The success rate of this kind of xenotransplantation was approximately 15-20%, similar to previous reports in the literature. Two xenotransplants (ER-PR-HER2-) were used; one at the 3^rd^ passage (P3887, PDX1), the other at the 4^th^ passage (Vari068, PDX2), both of which were derived from primary tumors of triple-negative breast cancer patients. The culture medium for different breast cell lines was illustrated in our previous reports [[1](#_ENREF_1), [2](#_ENREF_2)].

### Establishment of the PDX model

The PDX models were established in collaboration with Dr. Wicha’s group at University of Michigan, and the human tissues were utilized according to approved IRB protocols for research in human subjects. Three-week-old female NOD/SCID mice were obtained from Vital River Laboratory Animal Technology Company Limited and housed in AAALAC-accredited specific pathogen-free rodent facilities at University of Science and Technology of China.

### Flow-cytometry analysis and sorting

Each PDX model contained five to six tumors. To avoid the RNA amplification bias, we decided to use the PDX mixture to perform RNA-seq. Therefore, each group of the PDX model was equivalent to the average mixture including five or six biological replicates, meaning that we indeed used ten to twelve PDXs for this study. The samples were dissociated from the established PDXs, and then digested by collagenase into single-cell suspensions. The single-cell suspensions were incubated with anti-CD44, anti-CD24, and anti-Lineage mixed antibodies and the mouse cell antibody H2Kd (anti-H2Kd, anti-CD45, anti-235a, anti-CD31, and anti-CD140b; BD Pharmagen) as previously described [[3](#_ENREF_3)]. The ALDEFLUOR assay (Stem Cell Technologies) was performed following the manufacturer’s protocols. The cells were further analyzed for the expressions of ALDH and CD24/CD44. Four populations from the total tumor cells (ALDH^+^CD24^-^CD44^+^, ALDH^+^non-CD24^-^CD44^+^, ALDH^-^CD24^-^CD44^+^ and ALDH^-^non-CD24^-^CD44^+^) were sorted by MoFlo Astrios flow cytometry (Beckman Coulter). The negative controls of fluorescent activated cell sorting were done with previous standards [[2](#_ENREF_2)]. The cells were collected for further analysis.

### The sequencing library preparation

Total RNA of each group (in total 8 populations, 4 for each PDX) from the PDX was extracted using the RNeasy Micro Kit (QIAGEN) and RNase-Free DNase Set (QIAGEN) following the manufacture’s recommendations. We measured RNA concentration and quality by Agilent 2100 Bioanalyzer. Then the libraries were constructed by the RiboGone-Mammalian-Low Input Ribosomal RNA Removal Kit (Clontech) and NEBNext Ultra Directional RNA Library Prep Kit for Illumina (New England Biolabs). There were three technical replicates for each group, and we did not perform any extra amplification steps for total RNA (the lowest amount was about 50ng). The products were sequenced on Illumina Hiseq2500 platform using 2×50-bp SR reads.

### Short hairpin RNA plasmids and virus infection

Short hairpin RNA (ShRNA) plasmids were purchased from Sigma-Aldrich. The effective sequences of *PTGR1*, *P4HA2* and *RAB40B* were described in Additional file 1: Table S5. Briefly, the lentivirus was collected after 293T infection, and then the lentivirus infected stable SUM149 knockdown cell line was established after puromycin selection.

### MTT assay

Cells were seeded in 96-well plate at a density of 500 cells per well, and cultured for 3, 5, 7days. MTT (Biosharp) was added to the well each day, achieving a final concentration of 0.5mg/mL and then incubation in 37℃ for 4 hours. After adding the DMSO, the cell density was measured at OD490 with an Elx800 microplate reader (BioTek).

### Mammosphere formation assay

The cell line SUM149 was seeded at a density of 10000 cells/mL on Costar Ultra Low Attachment tissue culture plates. Mammospheres were cultured in MammoCult Human Basal Medium with added proliferation Supplement (Stem Cell Technologies). After 7 days, the size and number of mammospheres were determined using an inverted microscope. All experiments were done in triplicates.

### Quantitative real-time PCR (qRT-PCR)

The total RNA was extracted with RNAiso Plus (Takara), and the concentration was quantitated by Nanodrop (Thermo Fisher Scientific). The cDNA was reverse transcribed from 1ug RNA with HiScript II 1st Strand cDNA Synthesis Kit (Vazyme Biotech). qRT-PCR was perfomed using AceQ qPCR SYBR Green Master Mix (Vazyme Biotech) in a real-time PCR system (7300, Applied Biosystems). There were three replicates for each gene in parallel. BP (TATA-box binding protein) was used as a reference gene. The qRT-PCR primers were listed in Additional file 1: Table S1.

### Tumorigenicity in NOD/SCID mice

All mouse experiments were performed in accordance with Fudan University guidelines for the care and use of animals. For limiting dilution assay, 10000 and 1000 cells were injected into the fourth mammary gland of 4-week-old NOD/SCID mice. Tumors were monitored weekly until the diameter of tumors reached 1.0–1.5 cm. Tumor volume was calculated as 1/2 × length × width^2^.

### Analyses of RNA-seq data

The raw data of fastq format were aligned to the human reference genome (hg19, UCSC) with the TopHat version 2.0 [[4](#_ENREF_4)], and then assembled into transcripts with Cufflinks assembler version 2.2 [[5](#_ENREF_5)]. The transcripts of one group were integrated by Cuffmerge. The DEGs of pair-comparison were identified by Cuffdiff. The fragments per kilobase per million reads of genes in one PDX were normalized by Cuffnormal. The PCA was done with the FactoMineR package based on previous method [[6](#_ENREF_6)]. The GO and KEGG pathway analyses were done with DAVID 6.8 [[7](#_ENREF_7), [8](#_ENREF_8)], and then visualized by Apps ClueGO v2.3.2 of Cytoscape v3.4.0 [[9](#_ENREF_9), [10](#_ENREF_10)]. The Gene Set Enrichment Analysis (GSEA) was performed by GSEA v3.0 Beta [[11](#_ENREF_11), [12](#_ENREF_12)] with c2.cp.kegg.v6.0 and c5.all.v6.0 gene sets, in which number of permutations was set at 1000 and permutation type was applied with gene_set. We used WebGestalt [[13](#_ENREF_13), [14](#_ENREF_14)] to find the pathways related to the three prognostic genes.

## Results

### The expression of BCSC biomarkers ALDH, CD24 and CD44 in each sorted groups.

The expression of BCSC biomarkers ALDH and CD24/CD44 were as expected (Fig.1c). Groups B and D differed only in the enzyme activity of the biomarker *ALDH*, which has many isoforms. The expression of different ALDH isoforms might vary between different tumors. For instance, the isoforms *ALDH2*, *ALDH3A1*, *ALDH3A2*, *ALDH7A1* and *ALDH9A1* were highly expressed in the groups A and B from PDX1, while the isoforms *ALDH1A1*, *ALDH1A3*, *ALDH2*, *ALDH3A2* and *ALDH5A1* were highly expressed in the groups A and B from PDX2 (Fig.1c).

### The transcriptional differences between three states of BCSCs and the differentiated tumor cell population

To get the common DEGs in each state of BCSCs from the analyzed PDXs, we overlapped the DEGs of the pair-comparisons between each BCSC population and group D with fold change set at 1.2, based on the standard of our previous study [[2](#_ENREF_2)]. The A/D, B/D and C/D represented comparisons of three states of BCSCs, which were purified BCSCs which expressed both sets of BCSC markers, epithelial-like BCSCs and mesenchymal-like BCSCs, and differentiated tumor cells. The DEGs in A/D, B/D and C/D pair-comparisons were 3223, 3387 and 3065, respectively (Figure S1.a). To characterize the three states of BCSCs, we overlapped the DEGs of three pair-comparisons (Figure S1.b), and found that each state has its own unique DEG (Figure S1.b). For all states of BCSCs in common, there were 391 DEGs in the intersection set (Figure S1.b). These 391 genes were differentially expressed between BCSCs and differentiated tumor cells, so they revealed the common features in BCSCs. The Gene Ontology (GO) analysis based on biological process indicated that these genes were involved in cellular response to hypoxia, cell adhesion, extracellular matrix organization, cell cycle, etc (Additional file 2: Table S2).

### Compare each state of BCSCs with the differentiated tumor cell population

To characterize the exclusively transcriptional features of each state of BCSCs, we overlapped the DEGs of three pair-comparisons (Figure S1.b), and found that each state has its own unique DEGs (Figure S1.b). Comparing group A with D, there were 343 upregulated and 356 downregulated DEGs in the ALDH^+^CD24^-^CD44^+^ (group A) population. The GO analysis based on biological processes of these 699 DEGs showed that the upregulated DEGs participated in acitivation of phospholipase A2 activity, dTTP biosynthetic process, dolichol metabolic process, regulation of plasma membrane long-chain fatty acid transport, among other processes, while the downregulated DEGs participated in positive regulation of hh target transcription factor activity and endocardial cushion to mesenchymal transition involved in heart valve formation (Figure S1.c). Comparing group B with D, there were 511 upregulated and 456 downregulated DEGs in the ALDH^+^non-CD24^-^CD44^+^ (group B) population. The GO analysis based on biological process revealed that the upregulated DEGs corresponded to genes involved in urothelium development and rRNA 2’-O-methylation, while the downregulated DEGs participated in negative regulation of oocyte maturation and acetyl-CoA catabolic process (Figure S1.d). Additionally, there were other affected biological process, such as mRNA pseudouridine synthesis, receptor-mediated endocytosis involved in cholesterol transport, and transcription initiation from RNA polymerase I promoter for nuclear large rRNA transcript (Figure S1.d). Comparing group C with D, there were 395 upregulated and 421 downregulated DEGs in the ALDH^-^CD24^-^CD44^+^ (group C) population. The GO analysis based on biological process showed that these upregulated DEGs resembled those involved in regulation of peptidyl-tyrosine autophosphorylation, endocardial cushion fusion, oncostation-M-mediated signaling pathway, ATP generation from poly-ADP-D-ribose and so on, while the downregulated DEGs participated in negtive regulation of cell-cell adhesion mediated by cadherin, free ubiquitin chain polymerization and positive regulation of toll-like receptor 2 signaling pathway (Figure S1.e).

The GSEA was applied to identify the altered GO terms for each pair-comparison (A/D, B/D, and C/D). Among the unique 1412 DEGs between groups A and D, as well as the unique 1670 DEGs between groups B and D, there was no altered GO terms shared by analyzed PDXs. However, in the unique 1384 DEGs between groups C and D, there were 26 upregulated GO terms shared by analyzed PDXs, such as angiogenesis, regulation of cell adhesion, glycoprotein metabolic process, biological adhesion and response to oxygen containing compound, but no downregulated GO terms shared (Additional file 3: Table S3, the shared GO terms were marked by red; Additional file 1: Figure S2.a).

### Compare enriched epithelial-like BCSCs with enriched mesenchymal-like BCSCs

There were reciprocal expression patterns related to the epithelial-mesenchymal transition (EMT) and the mesenchymal-epithelial transition (MET) states between CD24^-^CD44^+^ population and the rest of the population or between ALDH^+^ population and ALDH^-^ population [[2](#_ENREF_2)]. We fully characterized these two states of BCSCs, enriched epithelial-like BCSCs and enriched mesenchymal-like BCSCs (group B marked by ALDH^+^non-CD24^-^CD44^+^ and group C marked by ALDH^-^CD24^-^CD44^+^), in the following differential expression analysis.

There were 4486 overlapped DEGs between groups B and C identified by the pair-comparisons (Figure S3.a). Then we identified 2805 out of the 4486 DEGs, the 1419 upregulated DEGs and 1386 downregulated DEGs in group B in common (Figure S3.b). Genes related to EMT state from previous study [[2](#_ENREF_2)] were screened out among the 2805 DEGs. The MET makers *CDH3*, *CLDN3*, *CLDN4*, *CLDN7* and *MKI67* were highly expressed in enriched epithelial-like BCSCs (group B), while the EMT markers *CDH2*, *FOXC2*, *MMP2*, *SNAI2* and *TWIST1* were highly expressed in enriched mesenchymal-like BCSCs (group C) (Figure S3.c). The GO analysis based on biological process showed that the upregulated DEGs in group B recapitulated those involved in mitotic nuclear division, chromosome organization and organelle organization, while the downregulated DEGs in group B were involved in regulation of cell motility, epithelial cell proliferation, cellular response to oxygen levels, cell-cell adhesion via plasma-membrane adhesion molecules, et.al (Figure S3.d), which was in accordance with previous reports that ALDH^+^ BCSCs are proliferative [[3](#_ENREF_3)], while CD24^-^CD44^+^ BCSCs are quiescent and invasive with low proliferative capacity [[2](#_ENREF_2)]. The cellular response to oxygen levels were downregulated in mesenchymal-like BCSCs, which is in accordance with previous reports that mesenchymal-like BCSCs prefer glycolysis and have lower ROS [[15](#_ENREF_15)], suggesting that different states of BCSCs may have different metabolic features. Furthermore, partially upregulated and downregulated DEGs mutually affected tissue development, regulation of the Wnt signaling pathway, regulation of transferase activity, et.al (Figure S3.d). The cellular plasticity between EMT and MET states in BCSCs may be associated with tumor invasion and metastasis.

To identify the altered GO terms between groups B and C, we also implemented the GSEA with the overlapped 4486 DEGs (Figure S3.b). There were 12 upregulated GO terms in group B from analyzed PDXs, involving in DNA packaging complex, nuclear nucleosome, chromatin silencing, regulation of gene expression epigenetic, protein DNA complex, chromatin assembly or disassembly, DNA conformation change, DNA packaging, gene silencing, protein hetero-tetramerization, and protein DNA complex subunit organization. In addition, there were also 354 downregulated GO terms in group B (upregulated in group C) shared by both PDXs, which were related to cell motility, cellular response, collagen binding, extracellular matrix, mesenchymal cell differentiation, regulation of cell adhesion, and regulation of epithelial to mesenchymal transition (Additional file 1: Figure S2.b, Additional file 3: Table S3).

We have previously revealed that ALDH^+^ or CD24^-^CD44^+^ populations display cellular plasticity [[2](#_ENREF_2)], which was able to transit into the other state, just as the cancer cell plasticity [[16](#_ENREF_16)]. Therefore, to eradicate BCSCs, it may be viable to synchronically target BCSCs in alternated states by a couple of biomarkers.

### Transcriptional analysis between ALDH^+^CD24^-^CD44^+^ BCSCs and the other three groups

To identify the DEGs in ALDH^+^CD24^-^CD44^+^ BCSCs, we compared group A with the other three groups with fold change set at 1.2 according to our previous standard [[2](#_ENREF_2)] in analyzed PDXs (Fig.2a). The numbers of intersected A/X (X stands for groups B, C or D) DEGs overlapped in analyzed PDXs were 3505 and 2360, respectively (Fig.2a). In theory, there should be one gene panel that was able to classify four groups of cells in all samples based on the four biomarker combinations. Therefore, we performed principal component analysis (PCA) to further distinguish group A from the other three groups in each PDX, trimming DEGs to 3105 and 1851 for PDX1 and PDX2, respectively (Fig.2b,c). Then we overlapped the trimmed DEGs of analyzed PDXs and identified 513 DEGs in the intersection set (Fig.2c). The hierarchical clustering of 513 DEGs showed that highly purified BCSCs (group A) differed from the other groups (Fig.2d). The GO analysis based on biological process of 513 DEGs showed that these genes mainly participated in regulation of cell differentiation, regulation of multicellular organismal development, cell migration, regulation of molecular function, etc (Fig.2e). The KEGG pathway analysis showed that the 513 DEGs participated in the p53 signaling pathway, signaling pathways regulating pluripotency of stem cells, and proteoglycans in cancer, basal cell carcinoma (Fig.2f, Additional file 4: Table S4). In addition, analysis of the KEGG pathway also showed that *FGFR2*, *EGFR*, *NTRK3*, *PGAM2*, *KIT*, *SLC7A5*, and *PIK3R1* participated in central carbon metabolism in cancer through the Warburg effect (Additional file 4: Table S4), supporting previous studies that CSCs prefer to utilize glycolysis as compared with differentiated cancer cells [[17](#_ENREF_17)].

We also applied the GSEA to identify the solely affected GO terms for ALDH^+^CD24^-^CD44^+^ BCSCs in analyzed PDXs. When comparing ALDH^+^CD24^-^CD44^+^ BCSCs with other three groups with all genes included, the solely upregulated GO terms shared was odorant binding, while no downregulated GO terms shared (Additional file 1: Figure S2.c, Additional file 3: Table S3), which might arise from the heterogeneity. When we used DEGs with fold change set at 1.2 to avoid noise from low-expressed genes to compare ALDH^+^CD24^-^CD44^+^ BCSCs with the others, there are no upregulated GO terms shared by analyzed PDXs, but six downregulated GO terms shared, which were related to the regulation of striated muscle cell differentiation, regulation of myotube differentiation, collagen trimer, central nervous system neuron development, proteoglycan metabolic process, and negative regulation of striated muscle cell differentiation (Additional file 3: Table S3, Additional file 1: Figure S2.c). When we performed GSEA with 3505 DEGs in PDX1 or 2360 DEGs in PDX2 (Fig.4a), there was no terms of GO and KEGG pathway shared by analyzed PDXs (Additional file 3: Table S4). In conclusion, via comparing group A (ALDH^+^CD24^-^CD44^+^ cell population) with the others by GSEA, we found that GO terms related to differentiation and development were significantly downregulated in ALDH^+^CD24^-^CD44^+^ BCSCs, which was in accordance with the above results.

### The relevant pathways of three prognostic genes

To find the relevant pathways of the three prognostic genes, we screened in the WebGestalt with KEGG, Reactome and Wikipathway databases. Firstly, *P4HA2* is related to arginine and proline metabolism, and metabolic pathways in KEGG database, besides collagen biosynthesis and modifying enzymes, collagen formation, and extracellular matrix organization in Reactome database, as well as amino acid metabolism in Wikipathway database. In breast cancer, *P4HA2* plays a role in extracellular matrix remodeling in low oxygen levels [[18](#_ENREF_18)], the overexpression of which promotes breast tumor progression [[19](#_ENREF_19)]. *P4HA2* is also a downstream target of p53, which participates in angiogenesis and tumor growth [[20](#_ENREF_20)]. Secondly, *PTGR1* is associated with synthesis of lipoxins, synthesis of leukotrienes and eoxins, arachidonic acid metabolism, and metabolism of lipids and lipoproteins in Reactome database, as well as NRF2 pathway in Wikipathway database. A previous study enunciated that NRF2 pathway is regulated by p53 pathway, especially for cell survival in the low level of ROS [[21](#_ENREF_21)]. Therefore, we speculate that the overexpressions of *P4HA2* and *PTGR1* might both mechanically affect p53 signaling pathway to hold the phenotype of ALDH^+^ CD24^-^CD44^+^ BCSCs.

Lastly, *RAB40B* is related to RAB geranylgeranylation, post-translational protein modification, and metabolism of proteins in Reactome database. The depletion of *RAB40B* is related to EMT and decreases breast cancer cell invasion [[22](#_ENREF_22)]. Low expressions of *RAB40B* was associated with decreased RFS in TNBC patients (n=255, p=0.0069). Therefore, the downregulation of *RAB40B* might be associated with tumor relapse, which requires further investigation. Based on all above, we speculate that *RAB40B* is related to the status of BCSCs by EMT transition, which was verified by the knockdown experiments that mesenchymal-like (CD24^-^CD44^+^) BCSCs were substantially increased, while epithelial-like (ALDH^+^) BCSCs were reduced. Taken together, the low expression of *RAB40B* could decrease mammosphere formation and tumor cell proliferation by probably reducing ALDH^+^ BCSCs, and the reason why *RAB40B* is associated with worse RFS might arise from the increased CD24^-^CD44^+^ BCSCs. Identifying the relationship between *RAB40B* and the different states of BCSCs, still requires further investigation.

## Supplementary Tables

### Table S1.The information of the sequencing data

| Sample | Intragenic Rate | Exonic Rate | Intronic Rate | Intergenic Rate | Split Reads | Expression Profiling Efficiency | Transcripts Detected | Genes Detected | Mapped rate |
| --- | --- | --- | --- | --- | --- | --- | --- | --- | --- |
| P1A-1 | 0.803 | 0.461 | 0.342 | 0.197 | 1,529,703 | 0.461 | 29,014 | 16,628 | 90.40% |
| P1A-2 | 0.803 | 0.461 | 0.342 | 0.196 | 1,541,924 | 0.461 | 29,016 | 16,642 | 90.40% |
| P1A-3 | 0.803 | 0.461 | 0.342 | 0.197 | 1,529,921 | 0.461 | 29,025 | 16,645 | 90.30% |
| P1B-1 | 0.815 | 0.385 | 0.43 | 0.185 | 1,180,951 | 0.385 | 28,738 | 16,463 | 90.70% |
| P1B-2 | 0.815 | 0.385 | 0.43 | 0.185 | 1,188,533 | 0.385 | 28,675 | 16,456 | 90.70% |
| P1B-3 | 0.815 | 0.385 | 0.43 | 0.185 | 1,180,115 | 0.385 | 28,723 | 16,455 | 90.70% |
| P1C-1 | 0.826 | 0.38 | 0.445 | 0.174 | 1,301,291 | 0.38 | 29,420 | 16,825 | 92.20% |
| P1C-2 | 0.825 | 0.38 | 0.445 | 0.174 | 1,307,904 | 0.38 | 29,387 | 16,814 | 92.20% |
| P1C-3 | 0.825 | 0.38 | 0.445 | 0.175 | 1,302,852 | 0.38 | 29,411 | 16,827 | 92.20% |
| P1D-1 | 0.805 | 0.417 | 0.388 | 0.195 | 1,465,464 | 0.417 | 29,322 | 16,749 | 89.30% |
| P1D-2 | 0.805 | 0.417 | 0.388 | 0.195 | 1,476,039 | 0.417 | 29,302 | 16,762 | 89.30% |
| P1D-3 | 0.804 | 0.417 | 0.388 | 0.195 | 1,465,035 | 0.417 | 29,365 | 16,795 | 89.20% |
| P2A-1 | 0.809 | 0.455 | 0.354 | 0.191 | 1,801,114 | 0.455 | 29,180 | 16,795 | 89.40% |
| P2A-2 | 0.809 | 0.455 | 0.354 | 0.191 | 1,812,013 | 0.455 | 29,189 | 16,775 | 89.30% |
| P2A-3 | 0.809 | 0.454 | 0.354 | 0.191 | 1,797,098 | 0.454 | 29,135 | 16,756 | 89.20% |
| P2B-1 | 0.834 | 0.505 | 0.329 | 0.166 | 1,691,800 | 0.505 | 28,133 | 16,084 | 88.30% |
| P2B-2 | 0.834 | 0.504 | 0.33 | 0.166 | 1,703,232 | 0.504 | 28,175 | 16,109 | 88.30% |
| P2B-3 | 0.834 | 0.504 | 0.33 | 0.166 | 1,694,346 | 0.504 | 28,137 | 16,095 | 88.30% |
| P2C-1 | 0.818 | 0.489 | 0.329 | 0.182 | 2,306,773 | 0.489 | 29,414 | 16,874 | 87.60% |
| P2C-2 | 0.818 | 0.489 | 0.329 | 0.182 | 2,322,093 | 0.489 | 29,384 | 16,909 | 87.60% |
| P2C-3 | 0.818 | 0.489 | 0.329 | 0.182 | 2,305,563 | 0.489 | 29,346 | 16,883 | 87.50% |
| P2D-1 | 0.805 | 0.483 | 0.323 | 0.194 | 1,874,271 | 0.483 | 28,804 | 16,543 | 87.00% |
| P2D-2 | 0.806 | 0.483 | 0.323 | 0.194 | 1,886,361 | 0.483 | 28,858 | 16,571 | 87.00% |
| P2D-3 | 0.805 | 0.483 | 0.323 | 0.194 | 1,871,908 | 0.483 | 28,848 | 16,561 | 86.90% |

* three replicates/group

### Table S5. PLKO.1 ShRNA sequences and qRT-PCR primers

| PLKO.1 ShRNA sequences | |
| --- | --- |
| PTGR1Sh | 5’-CTATCCTACTAATAGTGACTT-3’ |
| P4HA2Sh | 5’-GCAGTCTCTGAAAGAGTACAT-3’ |
| RAB40BSh-Sh2 | 5’-CCAGGATGATGCACGGCGGTT-3’ |
| RAB40BSh-Sh3 | 5’-CGACTCTTGGTAACATGAAAT-3’ |
| qRT-PCR Primers | |
| PTGR1-Fd | 5’-AGCACTTTGTTGGCTATCCTAC-3’ |
| PTGR1-Rv | 5’-CCCCATCATTGTATCACCTTCC-3’ |
| P4HA2-Fd | 5’-CAAACTGGTGAAGCGGCTAAA-3’ |
| P4HA2-Rv | 5’-GCACAGAGAGGTTGGCGATA-3’ |
| RAB40B-Fd | 5’-GTCCGGGCCTACGACTTTC-3’ |
| RAB40B-Rv | 5’-GGCCTGAAGTATCCCAGAGC-3’ |
| SOX2-Fd | 5’-GTCATTTGCTGTGGGTGATG-3’ |
| SOX2-Rv | 5’-AGAAAAACGAGGGAAATGGG-3’ |
| OCT4-Fd | 5’-CTTGCTGCAGAAGTGGGTGGAGGAA-3’ |
| OCT4-Rv | 5’-CTGCAGTGTGGGTTTCGGGCA-3’ |
| NANOG-Fd | 5’-AATACCTCAGCCTCCAGCAGATG-3’ |
| NANOG-Rv | 5’-TGCGTCACACCATTGCTATTCTTC-3’ |
| TBP-Fd | 5’-TGCACAGGAGCCAAGAGTGAA-3’ |
| TBP-Rv | 5’-CACATCACAGCTCCCCACCA-3’ |

## Supplementary Figures


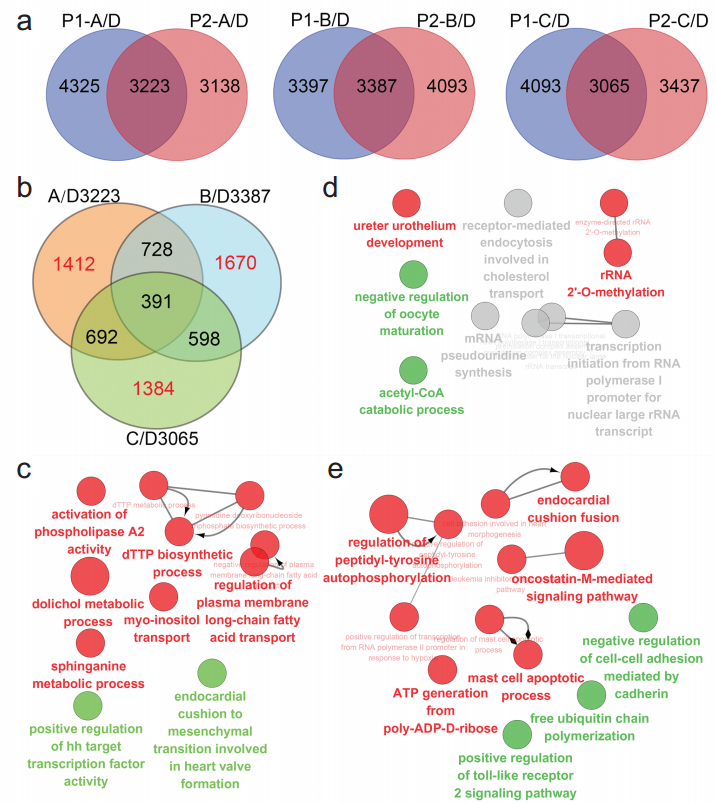


### Figure S1. Transcriptional comparison with each BCSC population to the differentiated tumor cell population.

(a) The Venn diagram of the DEGs between groups X (X stands for A, B and C ) and D. (b) The Venn diagram of the overlapped DEGs of three pair-comparisons identified in Fig.2A. The GO analysis based on biological processes of the DEGs visualized by Apps ClueGO v2.3.2 of Cytoscape v3.4.0 with network specificity set Detailed. (c) The DEGs between groups A and D. (d) The DEGs between groups B and D. (e) The DEGs between groups C and D. The red represented clusters inferred from upregulated DEGs. The blue represented clusters inferred from downregulated DEGs. The grey represented unspecific terms. The node size was related to the significance. The pathways with pV ≤ 0.05 were shown with ontology relations.


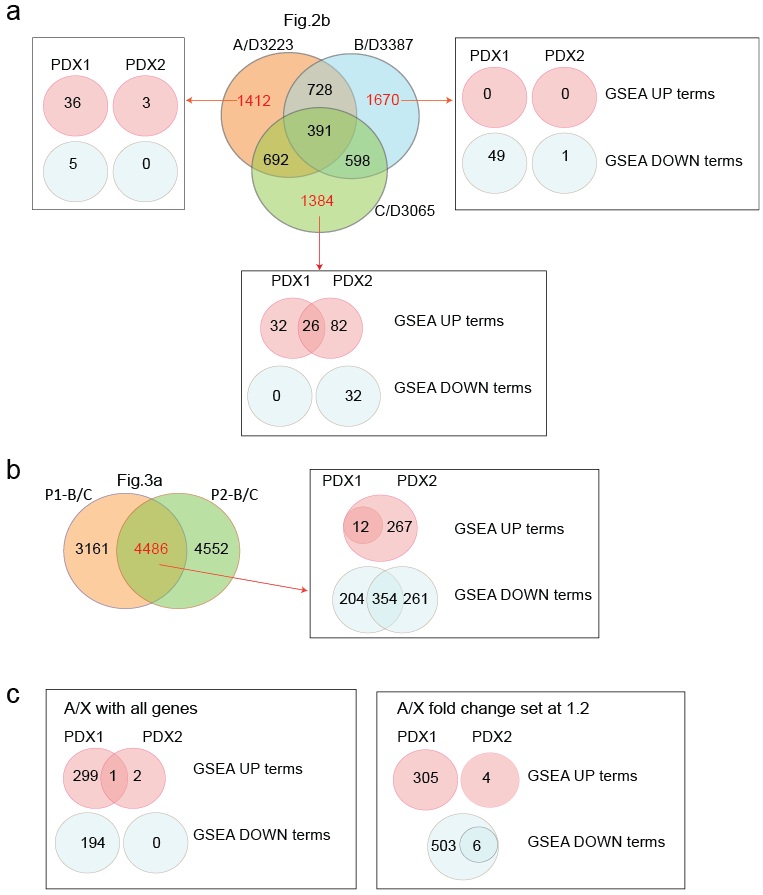


### Figure S2 (related to Figure S1 and S3). The visualized summary of GSEA results.

(a) In Figure 2B, we performed GSEA based on GO terms in 1412, 1670 and 1384 unique DEGs for comparisons A/D, B/D and C/D, respectively. There were no GO terms shared in comparisons A/D and B/D. In comparison C/D, there were 26 upregulated GO terms in groups C of analyzed PDXs, but none downregulated shared (Table S3, the shared terms were marked by red). (b) In Figure 3A, we performed GSEA based on GO terms in 4486 shared DEGs for comparison B/C. There were 26 upregulated GO terms and 354 downregulated GO terms in groups B of analyzed PDXs (Table S3, the shared terms were marked by red). (c) For comparing group A (ALDH^+^CD24^-^CD44^+^ BCSCs) with the other three groups, we used all genes from Cuffnormal to perform GSEA to find altered GO terms, which showed that there was only one upregulated GO term shared in groups A of analyzed PDXs (Table S3, the shared terms were marked by red). We also used the union set of DEGs with fold change set at 1.2 in each PDX (Figure 4A) to avoid noise from low-expressed genes to compare group A (ALDH^+^CD24^-^CD44^+^ BCSCs) with the others, which demonstrated that there were only six downregulated GO terms shared in groups A of analyzed PDXs. The pink and lightblue represent upregulated and downregulated terms in GSEA, respectively. In A/X, X stands for groups B, C or D.


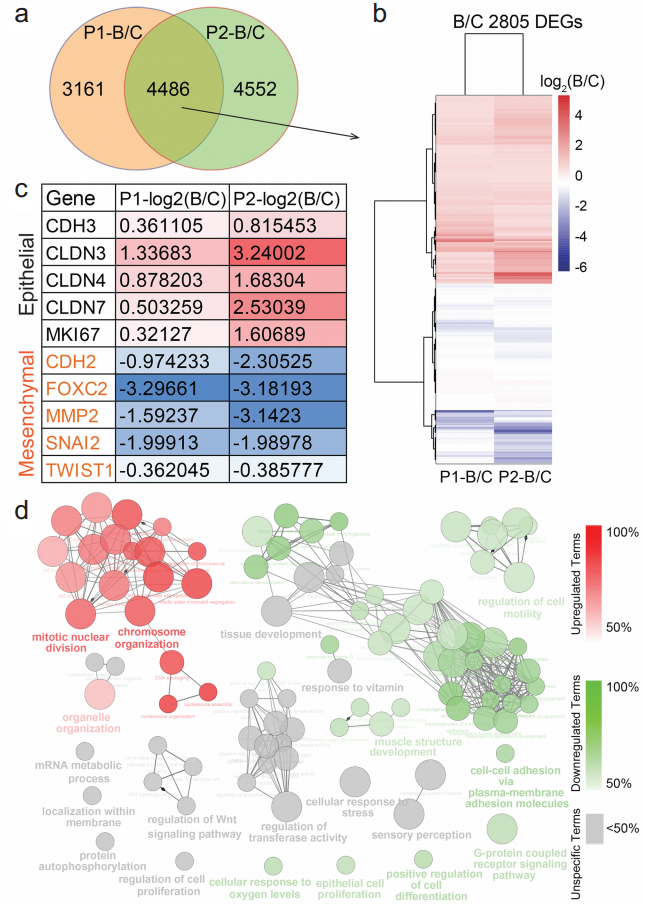


### Figure S3. Transcriptional comparison between enriched epithelial-like BCSCs and enriched mesenchymal-like BCSCs

(a) The Venn diagram of the DEGs between groups B and C with fold change set 1.2. (b) The upregulated and downregulated DEGs between groups B and C. (c) The DEGs between groups B and C involved in epithelial-mesenchymal transition. (d) The Go analysis based on biological process of the DEGs in Fig.3b visualized by Apps ClueGO v2.3.2 of Cytoscape v3.4.0 with network specificity set Medium.


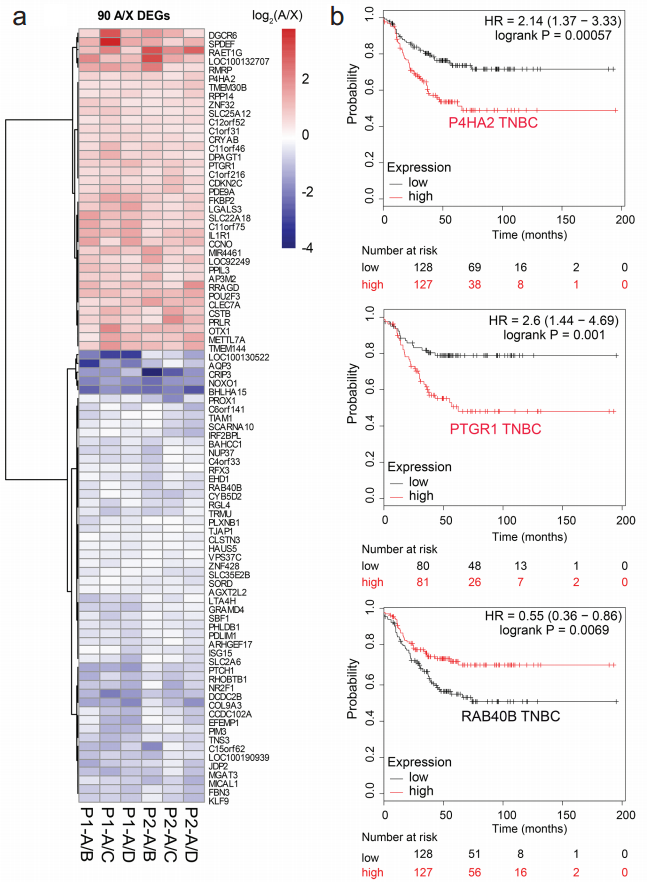


### Figure S4. The potential prognostic genes related to BCSCs in TNBC.

(a) The 90 unique DEGs of ALDH^+^CD24^-^CD44^+^ BCSCs in two PDXs. (b) The RFS of potential prognostic genes with different expressions in TNBC patients. X stands for groups B, C or D. TNBC, triple-negative breast cancer.


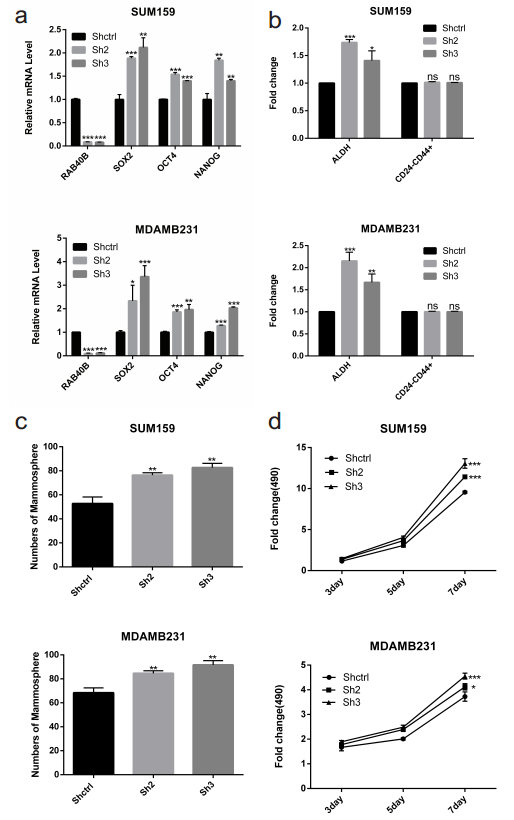


### Figure S5. The functional analysis of RAB40B in TNBC cell lines SUM159 and MDA-MB-231.

(a) The expressions of CSC-related genes in the RAB40B knockdown and the control (Shctrl) TNBC cell line SUM159 and MDA-MB-231. (b) The fold change for the proportion of each BCSC population in RAB40B-knockdown cells vs. Shctrl cells as assessed by fluorescent activated cell sorting. (c) The mammosphere formed in Shctrl cells and RAB40B-knockdown cells accessed by mammosphere formation assay. (d) The fold change for cell proliferation of RAB40B-knockdown cells vs. Shctrl SUM159 and MDA-MB-231 cells as assessed by MTT assay . *, P < 0.05; **, P < 0.01; ***, P <0.001; ns, not significant (compared with the corresponding Shctrl group). Error bars, mean± SD.

## References

1. Liu S, Ginestier C, Ou SJ, Clouthier SG, Patel SH, Monville F, et al. Breast Cancer Stem Cells Are Regulated by Mesenchymal Stem Cells through Cytokine Networks. Cancer Research. 2011;71:614-24.

2. Liu S, Cong Y, Wang D, Sun Y, Deng L, Liu Y, et al. Breast Cancer Stem Cells Transition between Epithelial and Mesenchymal States Reflective of their Normal Counterparts. Stem Cell Reports. 2014;2:78-91.

3. Ginestier C, Hur MH, Charafe-Jauffret E, Monville F, Dutcher J, Brown M, et al. ALDH1 Is a Marker of Normal and Malignant Human Mammary Stem Cells and a Predictor of Poor Clinical Outcome. Cell Stem Cell. 2007;1:555-67.

4. Kim D, Pertea G, Trapnell C, Pimentel H, Kelley R, Salzberg SL. TopHat2: accurate alignment of transcriptomes in the presence of insertions, deletions and gene fusions. Genome Biol. 2013;14:R36.

5. Trapnell C, Roberts A, Goff L, Pertea G, Kim D, Kelley DR, Pimentel H, Salzberg SL, Rinn JL, Pachter L. Differential gene and transcript expression analysis of RNA-seq experiments with TopHat and Cufflinks. Nat Protoc. 2012;7:562-78.

6. Treutlein B, Brownfield DG, Wu AR, Neff NF, Mantalas GL, Espinoza FH, Desai TJ, Krasnow MA, Quake SR. Reconstructing lineage hierarchies of the distal lung epithelium using single-cell RNA-seq. Nature. 2014;509:371-5.

7. Huang da W, Sherman BT, Lempicki RA. Systematic and integrative analysis of large gene lists using DAVID bioinformatics resources. Nat Protoc. 2009;4:44-57.

8. Huang da W, Sherman BT, Lempicki RA. Bioinformatics enrichment tools: paths toward the comprehensive functional analysis of large gene lists. Nucleic Acids Res. 2009;37:1-13.

9. Bindea G, Mlecnik B, Hackl H, Charoentong P, Tosolini M, Kirilovsky A, Fridman WH, Pages F, Trajanoski Z, Galon J. ClueGO: a Cytoscape plug-in to decipher functionally grouped gene ontology and pathway annotation networks. Bioinformatics. 2009;25:1091-3.

10. Shannon P, Markiel A, Ozier O, Baliga NS, Wang JT, Ramage D, Amin N, Schwikowski B, Ideker T. Cytoscape: a software environment for integrated models of biomolecular interaction networks. Genome Res. 2003;13:2498-504.

11. Mootha VK, Lindgren CM, Eriksson KF, Subramanian A, Sihag S, Lehar J, et al. PGC-1alpha-responsive genes involved in oxidative phosphorylation are coordinately downregulated in human diabetes. Nat Genet. 2003;34:267-73.

12. Subramanian A, Tamayo P, Mootha VK, Mukherjee S, Ebert BL, Gillette MA, Paulovich A, Pomeroy SL, Golub TR, Lander ES, Mesirov JP. Gene set enrichment analysis: a knowledge-based approach for interpreting genome-wide expression profiles. Proc Natl Acad Sci U S A. 2005;102:15545-50.

13. Zhang B, Kirov S, Snoddy J. WebGestalt: an integrated system for exploring gene sets in various biological contexts. Nucleic Acids Research. 2005;33:W741-W8.

14. Wang J, Duncan D, Shi Z, Zhang B. WEB-based GEne SeT AnaLysis Toolkit (WebGestalt): update 2013. Nucleic Acids Research. 2013;41:W77-W83.

15. Diehn M, Cho RW, Lobo NA, Kalisky T, Dorie MJ, Kulp AN, et al. Association of reactive oxygen species levels and radioresistance in cancer stem cells. Nature. 2009;458:780-3.

16. Meacham CE, Morrison SJ. Tumour heterogeneity and cancer cell plasticity. Nature. 2013;501:328-37.

17. Ciavardelli D, Rossi C, Barcaroli D, Volpe S, Consalvo A, Zucchelli M, et al. Breast cancer stem cells rely on fermentative glycolysis and are sensitive to 2-deoxyglucose treatment. Cell Death Dis. 2014;5:e1336.

18. Gilkes DM, Bajpai S, Chaturvedi P, Wirtz D, Semenza GL. Hypoxia-inducible factor 1 (HIF-1) promotes extracellular matrix remodeling under hypoxic conditions by inducing P4HA1, P4HA2, and PLOD2 expression in fibroblasts. J Biol Chem. 2013;288:10819-29.

19. Xiong G, Deng L, Zhu J, Rychahou PG, Xu R. Prolyl-4-hydroxylase alpha subunit 2 promotes breast cancer progression and metastasis by regulating collagen deposition. BMC Cancer. 2014;14:1.

20. Teodoro JG, Parker AE, Zhu X, Green MR. p53-mediated inhibition of angiogenesis through up-regulation of a collagen prolyl hydroxylase. Science. 2006;313:968-71.

21. Chen W, Jiang T, Wang H, Tao S, Lau A, Fang D, Zhang DD. Does Nrf2 contribute to p53-mediated control of cell survival and death? Antioxid Redox Signal. 2012;17:1670-5.

22. Jacob A, Jing J, Lee J, Schedin P, Gilbert SM, Peden AA, Junutula JR, Prekeris R. Rab40b regulates trafficking of MMP2 and MMP9 during invadopodia formation and invasion of breast cancer cells. J Cell Sci. 2013;126:4647-58.
